# An automated neural network-based stage-specific malaria detection software using dimension reduction: The malaria microscopy classifier

**DOI:** 10.1016/j.mex.2023.102189

**Published:** 2023-04-20

**Authors:** Preißinger Katharina, Kézsmárki István, Török János

**Affiliations:** a Department of Applied Biotechnology and Food Sciences, BME, Budapest 1111, Hungary; b Research Center for Natural Sciences, Institute of Enzymology, Budapest 1111, Hungary; c Department of Physics, BME, Budapest 1111, Hungary; d Department of Experimental Physics V, University of Augsburg, Augsburg 86159, Germany; e Department of Theoretical Physics, Institute of Physics, BME, Műegyetem rkp. 3, Budapest H-1111, Hungary; f MTA-BME Morphodynamics Research Group, BME, Budapest 1111, Hungary

**Keywords:** The malaria stage classifier, Malaria, Red blood cells, Malaria diagnosis, Artificial intelligence, Cell detection, Software

## Abstract

Due to climate change and the COVID-19 pandemic, the number of malaria cases and deaths, caused by the *Plasmodium* genus, of which *P. falciparum* is the most common and lethal to humans, increased between 2019 and 2020. Reversing this trend and eliminating malaria worldwide requires improvements in malaria diagnosis, in which artificial intelligence (AI) has recently been demonstrated to have a great potential. One of the main reasons for the use of neural networks (NNs) is the time saving through automatising the process and the elimination of human error. When classifying with two-dimensional images of red blood cells (RBCs), the number of parameters fitted by the NN for the classification of RBCs is extremely high, which strongly influences the performance of the network, especially for training sets of moderate size. The complicated handling of malaria culturing and sample preparation does not only limit the efficiency of NNs due to small training sets, but also because of the uneven distribution of red blood cell (RBC) categories. To boost the performance of microscopy techniques in malaria diagnosis, our approach aims at resolving these drawbacks by reducing the dimension of the input data and by data augmentation, respectively. We assess the performance of our approach on images recorded by light (LM), atomic force (AFM), and fluorescence microscopy (FM). Our tool, the Malaria Stage Classifier, provides a fast, high-accuracy recognition by (1) identifying individual RBCs in multi-cell microscopy images, (2) extracting characteristic one-dimensional cross-sections from individual RBC images. These cross-sections are selected by a simple algorithm to contain key information about the status of the RBCs and are used to (3) classify the malaria blood stages. We demonstrate that our method is applicable to images recorded by various microscopy techniques and available as a software package.

• Identifying individual RBCs in multi-cell microscopy images.
• Extracting characteristic one-dimensional cross-sections from individual RBC images. These cross-sections are selected by a simple algorithm to contain key information about the status of the RBCs and are used to.
• Classify the malaria blood stages. We demonstrate that our method is applicable to images recorded by various microscopy techniques and available as a software package.

## Graphical abstract

**Figure:**
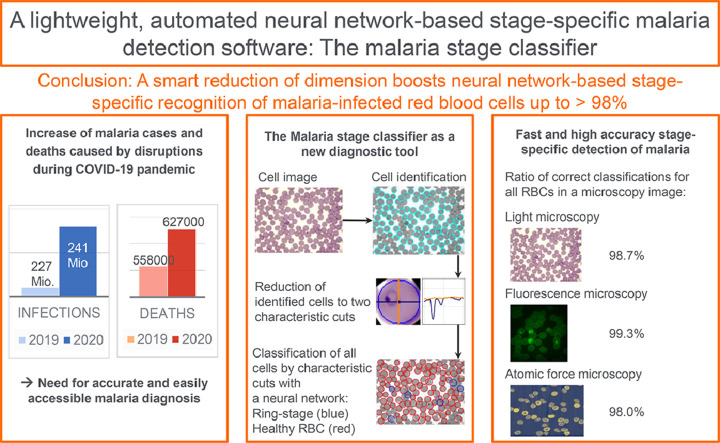

Specifications Table

**Table:** 

Subject area:	Medicine and Dentistry
More specific subject area:	Biophysics, Diagnostics
Name of your method:	The malaria stage classifier
Name and reference of original method:	N.A.
Resource availability:	Software: https://github.com/KatharinaPreissinger/Malaria_stage_classifier Documentation: https://malaria-stage-classifier.readthedocs.io/en/latest/index.html Data set: https://zenodo.org/record/6866337 Equipment: • Albumax, 25 mg/L Gentamycin • RPMI 1640 • VWR microscope slides (90° ground edges, nominal thickness 0.8 - 1.0 mm), • MFP-3D AFM (Asylum Research, Oxford Instruments), • OTESPA-R3 from Bruker with a rectangular shape, a tip radius of 7–10 nm, a spring constant of 26 N/m, operated at a frequency of 280 - 300 kHz with a drive amplitude of 250 - 300 mV • AFM acquisition software: Igor Pro 6.37 • Olympus IX81 inverted microscope • OMICRON (Rodgau-Dudenhofe, Germany) LaserHub (405 nm 120 mW CW diode, 488 nm 200 mW CW diode, 561 nm 156 mW CW diode, 642 nm 140 mW CW diode) • OMICRON Control centre software (v.3.3.19) • EM-CCD camera (Andor iXon DU-885KCSO-VP, Oxford instruments) • emission filter set (TRF89901-EM-ET-405/488/561/640, Chroma Technology, Bellows Falls, VT USA) • coverslips (nominal thickness 150 μm)

## Method details

### Design and implementation of the malaria stage classifier

The Malaria Stage Classifier is designed to facilitate and accelerate the staging of malaria infected RBCs in microscopy images. Due to its robustness against imaging platform-specific features, it is applicable to a wide range of light microscopy images. The interface of the application is arranged in tabs, which makes it easy to follow the image processing steps. The Malaria Stage Classifier further offers the possibility to manually optimise the cell detection and classification*.*

### Data set

**Fig. 1:**
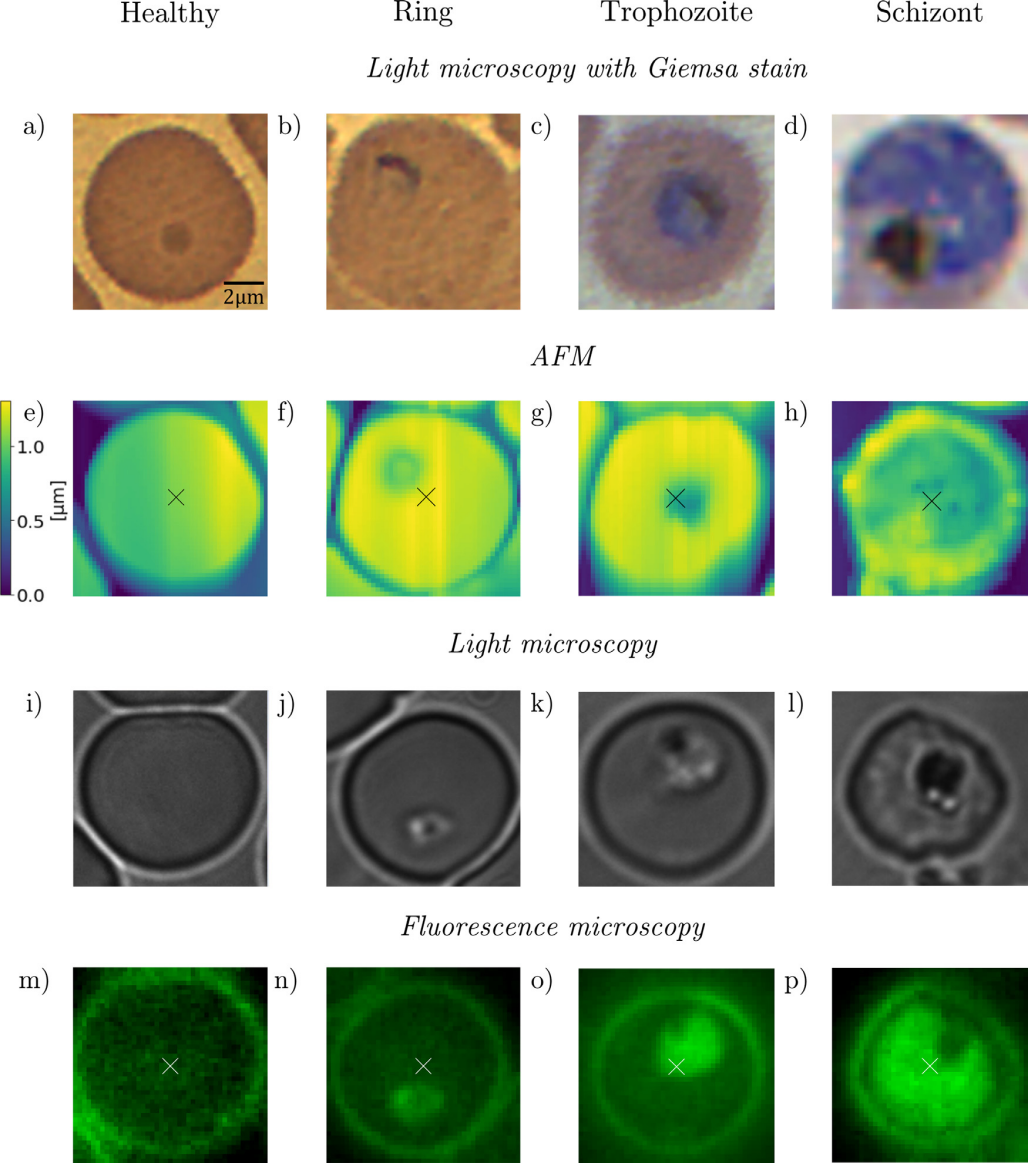
Representative microscopy images of healthy RBCs and RBCs infected with ring-, trophozoite, and schizont-stage parasites. First and second row: Light microscopy images of Giemsa-stained RBCs with corresponding AFM images. Third and fourth row: Light microscopy images of a different set of RBCs and their fluorescence maps after excitation at 405 nm.

For training and testing of our method, we used RBC images from our previous study [1]. The dataset contains images of red blood cells infected malaria. We chose *P. falciparum* cultures because it is the most common and lethal variant amongst human Plasmodium species, that is dominant in various regions. We used three different imaging techniques to test the robustness of our method. We used optical microscopy images of Giemsa-stained RBCs, the AFM images were measured on unstained thin blood films of *P. falciparum* cultures, revealing the structural changes during the malaria blood cycle. Similarly, the fluorescence signals were obtained exciting the RBCs by light of 405 nm wavelength. For more details, see the supplementary details section. From the measurements, we obtained 173 AFM images, 1232 fluorescence microscopy images, and 792 Giemsa-stained light microscopy images [747 from [2] and 45 recorded by us]. The images were labelled by three microscopists after staining each microscopy slide with Giemsa's stain. In case of the fluorescence and atomic force microscopy, we did not stain the cells prior to imaging in order to avoid changes in the cell shape. However, for the labelling performed after image acquisition, we stained the RBCs, which allowed us to identify both, the species and the parasite. With the cell images, we build a database by extracting and manually classifying single RBC images from the measurement images mentioned above. This classification forms the basis for the stage-specific detection algorithm presented in this paper. Fig. 1 shows representative images of the malaria blood stages for each imaging method.

The distribution of the stage categories included in the data set is shown in Table 1.

**Table 1:** Distribution of healthy RBCs and RBCs infected by the different malaria stages in the whole set of single-RBC images.

Imaging method	*Total*	*Healthy*	*Ring*	*Trophozoite*	*Schizont*
LM	26,223	24,586	502	575	560
AFM	7386	5679	350	558	799
FM	45,726	40,874	1946	1598	1265

For the evaluation of the AFM images, we used the software Igor Pro, which only allows exporting images as text files. Therefore, our algorithm is designed to handle text and image files as input.

### Data loading

For processing the extracted RBC images, either as text or image files, we converted them to greyscale in order to extract cuts characteristic to the malaria stages, as will be explained in “Reduction of dimensionality as a tool for feature selection in malaria-infected RBC”. In our case the information contained in the grayscale image is enough for the classification of malaria stages. Using a colour image would significantly increase the number of inputs. Due to the curse of dimensionality, which describes the problem of overfitting when using a large dimensional vector, our network would require a higher number of parameters. This not only results in the need for a large set of training data but will also lead to overfitting. Due to the strong contrast between cell and parasite, the conversion did not influence the classification accuracy. In case of atomic force and some light and fluorescence microscopy images, the contrast between background and RBCs is not strong enough to locate single cells, which impacts the accuracy of the detection. Hence, the images are binarised based on pixel intensities by Otsu's method [3]. While the processing of atomic force microscopy images requires an additional step, it is sufficient to enhance brightness, sharpness, and contrast in the light and fluorescence microscopy images. Python offers a module for automatic enhancement of images by a manually chosen factor [4], which can be applied to highlight the RBCs in contrast to the image background. The processed images are then used for cell detection, employing the Hough gradient method. While the detection parameters are preset, they can be manually adjusted by the user.

### Segmenting and image processing

**Fig. 2:**
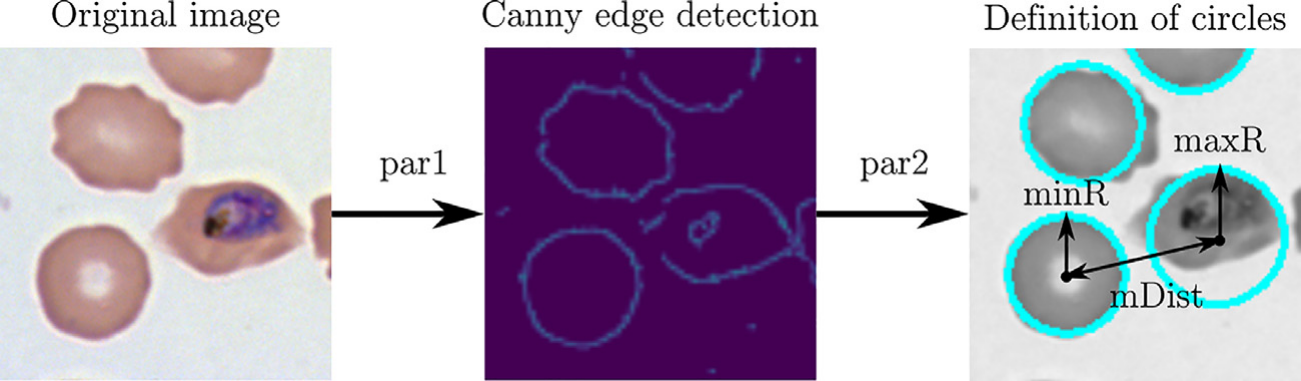
Graphical representation of the Hough circle method. par1 is used to define the threshold for the Canny edge detector, while par2 denotes the threshold for the centre detection. mDist, minR, and maxR are shown in the right image.

The RBCs in the microscopy images are detected in three steps: localising edges, finding the centre of the object, and calculating its radius. This approach is also known as the Hough circle method [5]. The method is controlled by five parameters, *mDist, par1, par2, minR,* and *maxR. mDist* defines the minimum pixel distance between the centres of two objects. *par1* is the threshold value for edge detection by the Canny edge detector [6]*. par2* sets the threshold for the number of edge points to declare the object a circle*. minR* and *maxR* set the minimum and maximum size of the radius in pixels. Fig. 2 shows a graphical representation of the Hough circle method. We tested various values for the five parameters on the RBC images and determined how many cells were detected in the images. The values for each parameter, which showed the best result are shown in Table 2 [7] and allow for the localisation of more than 95% of all RBCs (approx. 7000 for each imaging technique).

**Fig. 3:**
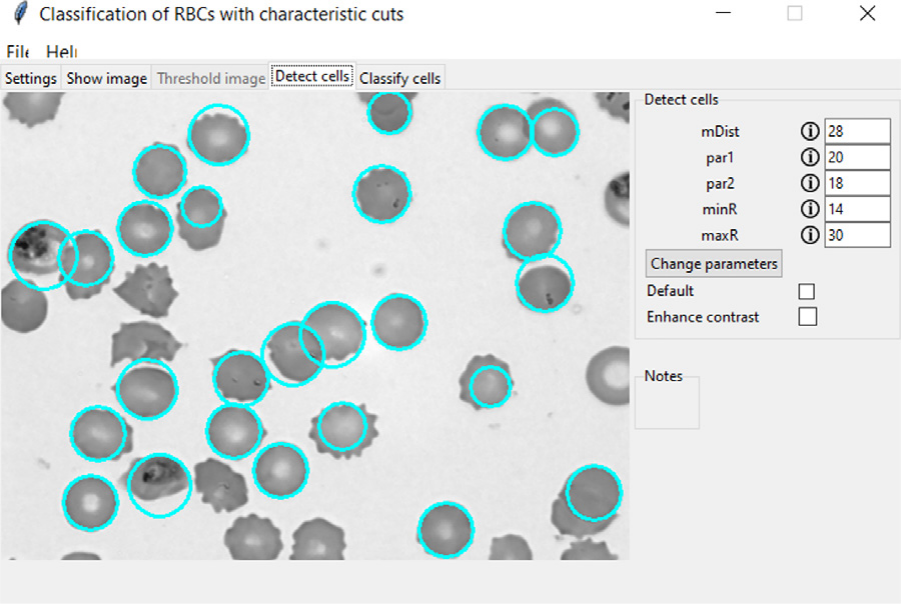
Cell detection in light microscopy image. The detected cells are marked with a cyan circle.

**Table 2:** Optimum values for the sensitivity of the Hough gradient method for various imaging methods with the probability of cell detection.

Imaging method	*mDist [px]*	*par1*	*par2*	*minR [px]*	*maxR [px]*	Detection probability
LM	28	20	18	14	30	95%
AFM	28	10	18	14	30	96%
FM	28	20	18	14	30	97%

We note that limitations can occur for not circular or overlapping RBCs, which can be handled by manually adjusting the detection parameters. The centre and radius of the cells, detected by the Hough gradient method, define the contour and image coordinates associated with the RBCs, which are used for the calculation of the geometric and gravitational centre. While the optimum values work for the tested images, we added the option to manually adjust each parameter in order to improve the cell detection. Fig. 3 shows a representative microscopy image after running the cell detection algorithm with the default parameters.

## Reduction of dimensionality as a tool for feature selection in malaria-infected RBC

**Fig. 4:**
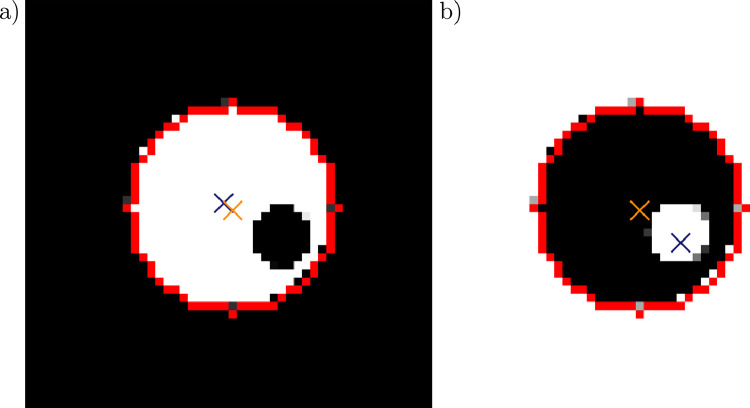
Location of the geometric and gravitational centre in simple cylinders with holes. Geometric and gravitational centre are shown as an orange and blue cross. The effect of the hole on the location of the gravitational centre is shown for a black (a) and white cylinder.

The accurate detection of the malaria blood stage plays a crucial role in diagnosis. Therefore, we tried to find a suitable measure for the characteristic features of the intra-erythrocytic stages, which should be insensitive to external noise, often present in AFM and fluorescence microscopy images due to tip contamination or background illumination. We determined characteristic cuts through RBC images by two measures: the geometric centre and the centre of gravity, which are defined by:

$${\vec{r}}_{geo}=\frac{1}{n}\sum_{i,j}{\vec{r}}_{i,j}$$

$${\vec{r}}_{grav}=\frac{1}{n}\sum_{i,j}h\left(\;{\vec{r}}_{i,j}\right)\;{\vec{r}}_{i,j}$$

where $h(\;{\vec{r}}_{i,j})$ denotes the height (AFM) or intensity (fluorescence and light microscopy) of a pixel at position ${\vec{r}}_{i,j}$. The coordinates inside the RBCs are calculated form the circle fit of the contour, which is returned by the Hough gradient method. The idea behind this approach is to localise the parasites inside the cell, i.e. the coordinates are weighted by their corresponding height or intensity values. Fig. 4 shows the effect of a region with lower intensity on two images of cylinders. The presence of the hole inside the cylinders leads to a shift of the gravitational centre, either away or towards the hole, which is indicated by an arrow. In case of the dark cylinder, the shift is higher, as the image values of black pixels are 0 and the values of white pixels 255. Therefore, the presence of the white hole has a larger effect on the position of the gravitational centre. Similarly, this shift can be observed for RBC images. From this follows that the height or intensity values along the straight line through both centres represent a cross-sections of the parasite. In the further course of the paper, this straight line is defined as the parasite cut.

**Fig. 5:**
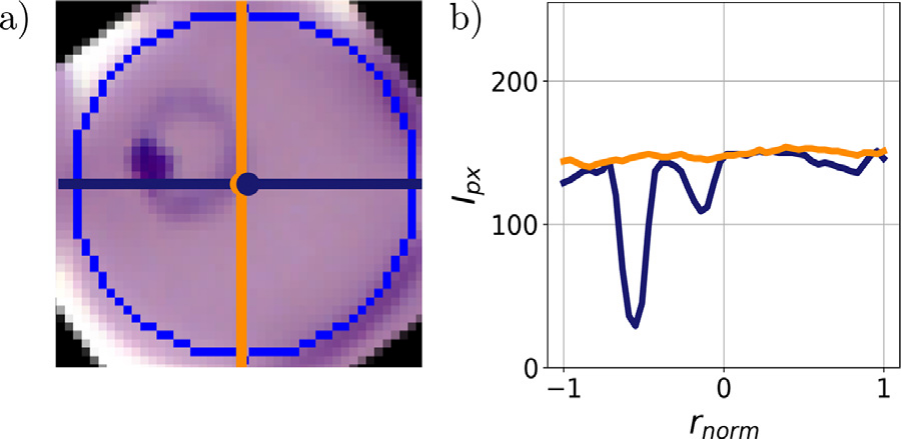
Characteristic cuts of a parasite. (a) Light microscopy image showing the parasite cut (blue) and the additional cut spanning 90° (orange) with it. (b) Corresponding intensity profiles. The contour of the cell is shown in blue.

An evaluation of the efficiency of the presented measure is shown in our paper about the stage-specific detection of malaria-infected RBCs based on dimension reduction [1]*.* The efficiency was tested on at least 350 ring, 550 trophozoite, and 550 schizont-stage parasites. In more than 50% of the cases, the parasite cut captures the ring-stage parasite, while the detection probability of the trophozoite-stage parasite is > 85%. In the schizont-stage, the parasites typically fill up most of the RBC. Therefore, the characteristic cuts going through the cell always intersect the parasite, leading to a detection probability of 100%. To fully represent the characteristic features of single cells, the parasite cut is supplemented by an additional cut, spanning 90° with it. A representative image is shown in Fig. 5*.*

**Fig. 6:**
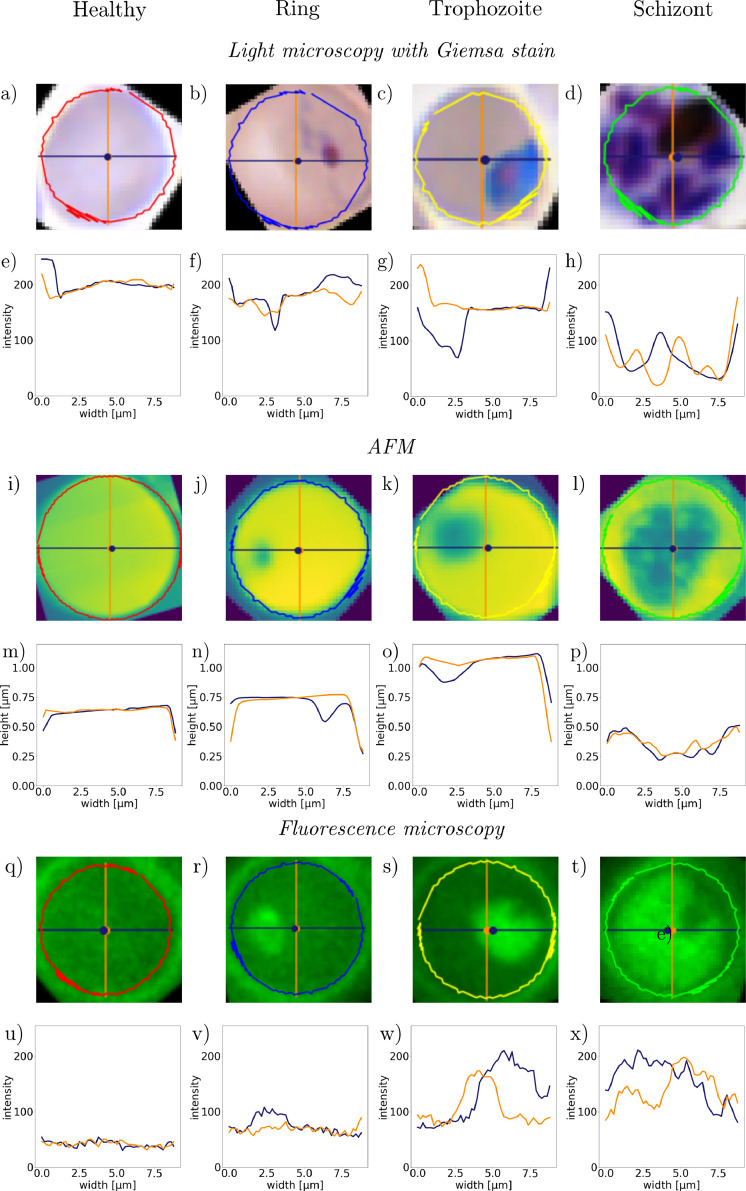
Characteristic cuts of malaria-infected RBCs imaged with atomic force (a) – (h), fluorescence (i)-(p), and light microscopy (q)-(x).

The dimensionality reduction revealed similar features for all three imaging techniques, as shown in Fig. 6. While the cuts through healthy cells show flat profiles, the presence of the parasite significantly changes the height and intensity profile measured by atomic force, light, and fluorescence microscopy. In our previous paper [1], we observed that the difference between the two profiles plays an important role in the classification of the malaria stages.

**Fig. 7:**
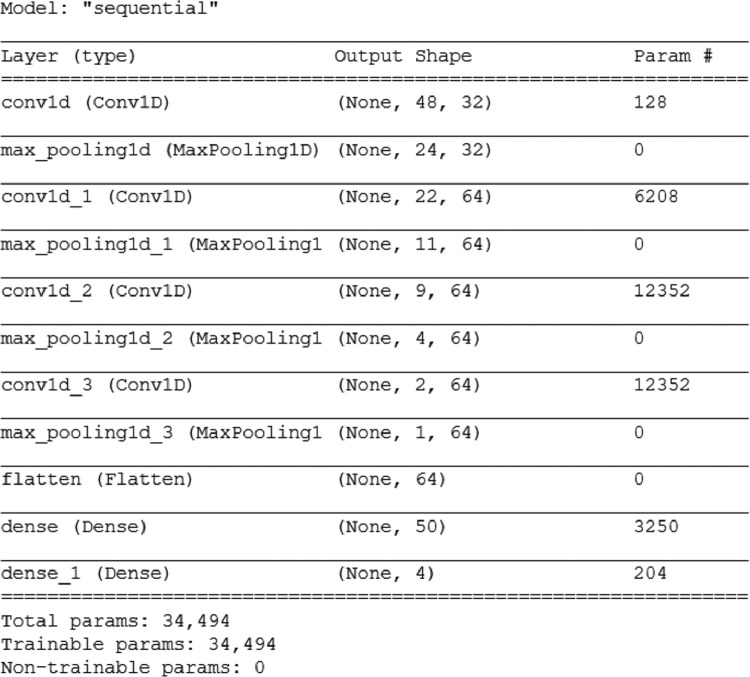
Neural network architecture. The network is used for the stage-specific classification of RBCs.

## Neural network architecture

When dealing with images, the most suitable models are convolutional neural networks (CNN). Generally, they contain convolutional, pooling, and dense layers [8], which we combined in such a way as to fulfil the following conditions: applicability to the stage-specific cuts, classification into four categories: healthy, ring, trophozoite, schizont, minimum performance of > 90%, smallest possible amount of fit parameters to guarantee short computation time. In automatic diagnosis, the major challenge is the variation of staining protocols, which prohibits the use of the same trained network for all applications. Therefore, we decided to go for a simple CNN with a small number of layers instead of using MobileNet or ResNet for our stage-specific classification. To test the various architectures of the NNs presented in Table 3, we used the full set of single RBC images (see Table 1). Caused by the measurement technique, our data set was imbalanced. We equalized the number of images for each category by rotation, shearing, and reflection of the single cell images, resulting in a total number of ∼68,000 AFM. The augmentation algorithms are functions provided by the OpenCV library (OpenCV, 2022). Each augmentation is done by randomly selecting the parameters, e.g. generating a random number for the rotation angle. For a representative result, all models were run for 20 epochs. In each model, the convolutional layers are followed by a pooling layer and two fully-connected layers at the end of the network. Our input images were split into a random training and test set at a ratio of 90% to 10%. We tested various numbers of feature maps and convolutional layers based on the number of parameters fitted by the neural network, ranging from 30,000 to 120,000. Table 3 shows a selection of these networks with the most promising performances and the corresponding number of feature maps.

**Table 3:** Classification accuracy on a test set of RBCs images for various network architectures.

Network	Feature map	Performance	Parameters
M1	4 × 32	80%	81,566
M2	2 × 32, 2 × 64	87%	68,446
M3	4 × 64	92%	114,878
M4	1 × 32, 3 × 64	96%	34,494

Model M4 provides the best compromise between the amount of fit parameters and classification accuracy. As the network was prone to overfitting, we added the kernel regulariser *L2* with a hyperparameter of 0.001 to the first fully-connected layer. The complete architecture of the NN for a one-dimensional input, which is used for the classification of the single RBC in microscopy images, is given in Fig. 7*.*

Our tests on various architectures of NNs show that network M4 performs best on the test data, reaching 96% accuracy. Therefore, we chose this architecture to test the classification accuracy in dependence of the imaging techniques on two-dimensional images and their corresponding characteristic cuts.

## Method validation

In the previous sections, we have described the methods, which form the main parts of the Malaria Stage Classifier. To find suitable parameters for the presented methods, we tested performance of the network (M4), which was implemented in the software package, for all microscopy techniques, first on two-dimensional single RBC images and then on characteristic cuts through the images capturing the most significant features of the malaria blood stages, as they represent the intensity (in case of light and fluorescence microscopy images) and height profile (AFM images) of the cell stages (see Fig. 5). Our input data set contained RBC images acquired with light, atomic force, and fluorescence microscopy. To balance the distribution of healthy cells and the malaria blood stages, we augmented the data set by rotation, shearing, and reflection, leading to a collection of ∼98,000 LM, ∼68,000 AFM, and 163,000 FM images and an equal number of characteristic cuts for each imaging method, respectively. The characteristic cuts are fed to the NN as a one-dimensional array, normalised to a length of 50 data points.

### Performance of the neural network on the characteristic cuts

**Fig. 8:**
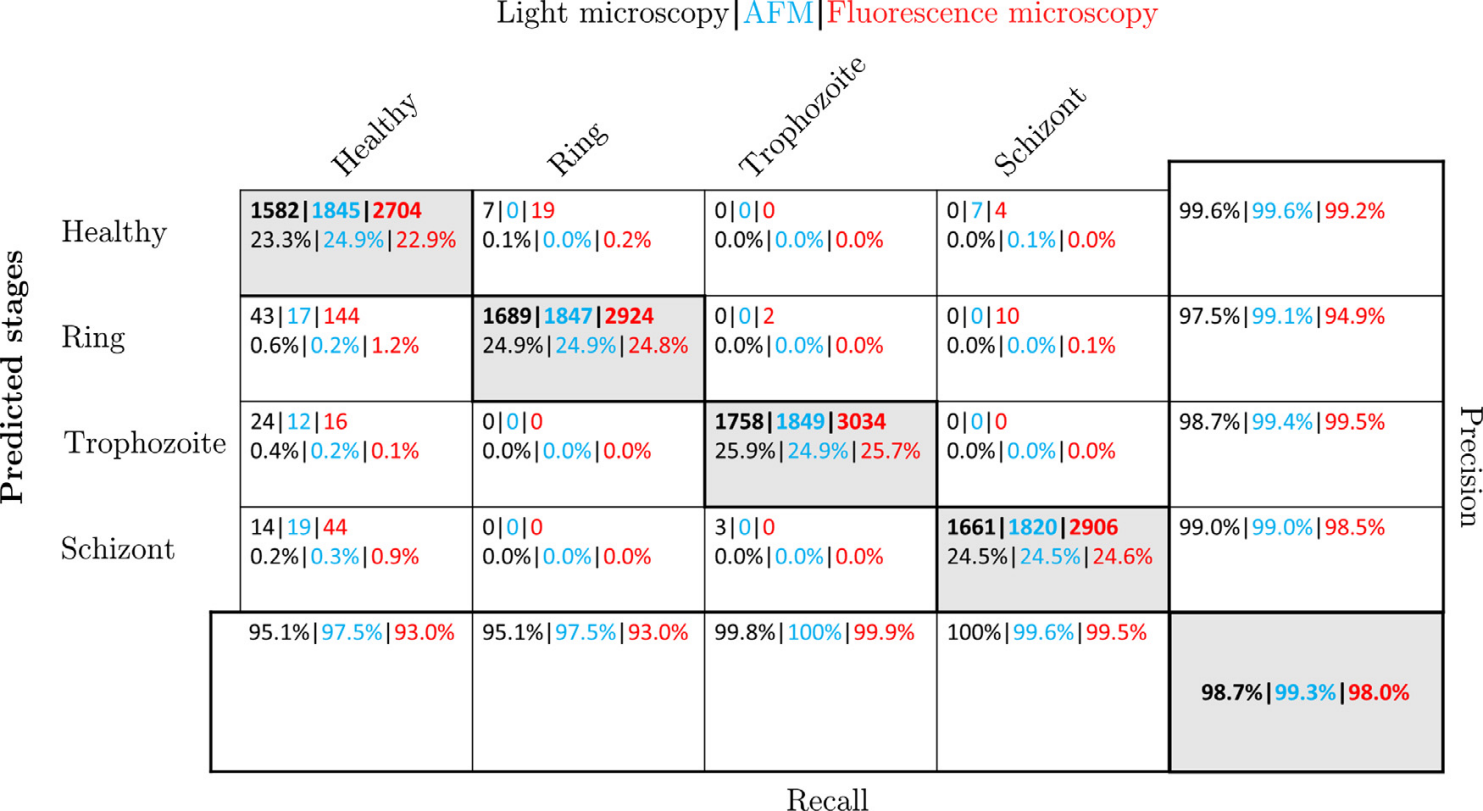
Performance of the NN on characteristic cross-sections. The classification results on the test set, as obtained on the characteristic cuts through light microscopy (black), AFM (blue) and fluorescence microscopy (red) images, are respectively summarised in the confusion matrix. The labels of the rows are the categories predicted by the NN-based classifier, while the labels of the columns indicate the classification by human experts. The diagonal elements show the correctly predicted cells, while the off-diagonal elements correspond to false classifications. The last column shows the precision for each category and the recall is shown in the bottom row. Each field displays the number of counts and the corresponding percentage with respect to the total number of cells in the test set. The overall classification performance is displayed in the grey field in the bottom right corner.

To predict the intra-erythrocytic stages of malaria, our set of images and specific cuts was split into a random 90%−10% train-test data set split. The training and test set for light microscopy contained RBC images of 17 malaria-infected patients from data published by Abbas et al. [2]*.* As described in the previous section, we trained our network for 20 epochs. In our previous publication [1], we show that we were able to reach 86–93% accuracy on the two-dimensional RBC images. By reducing the input dimension, we reached an even higher performance, when feeding the set of characteristic cuts to the network. The detailed results are shown in Fig. 8 in form of a confusion matrix, where *precision* and *recall* are defined as

$$precision=\frac{C_{ij}}{\sum_{j=1}^{N}C_{ij}}$$

$$recall=\frac{C_{ii}}{\sum_{i=1}^{N}C_{ij}}.$$

**Fig. 9:**
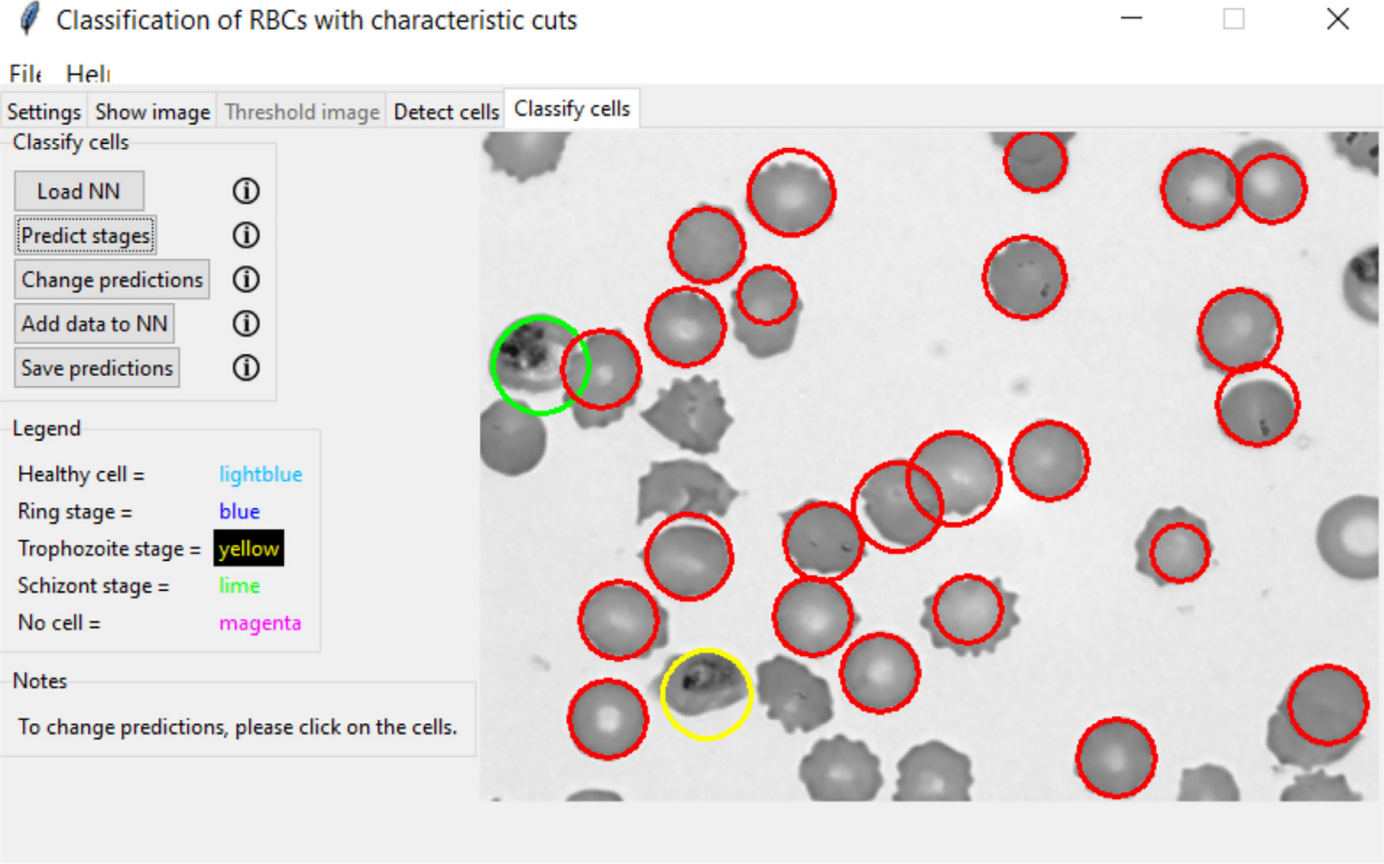
Classification of RBCs in a thin blood film. The image shows a thin blood film of a malaria-infected patient [2].

The overall accuracy of the classification, as shown in the large grey box, is calculated as the ratio of correct predictions, independent of the categories, to the total of values in the confusion matrix [9]

$$accuracy=\frac{\sum_{i=1}^{N}C_{ij}}{\sum_{i=1}^{N}\sum_{j=1}^{N}C_{ij}}$$

where *N* the number of classes*, i* the row index and *j* the column index. The detailed numbers of the classification are shown for each of the predicted (rows) and manually labelled stages (columns). With the characteristic cuts as input, our NN reaches more than 98% accuracy on the test sets, irrespective of the imaging method, outperforming the two-dimensional images. While our method has limitations, when the contrast of the images is very low, it still works uniformly well for all stages and imaging methods. We also observed a significant reduction in computation time by a factor of 18 to only a few milliseconds [1]. Together with the cell detection algorithm, the pre-trained networks for each imaging method form the main elements of the Malaria Stage Classifier. In Fig. 9, a sample image of a successful classification of infected RBCs is shown. Healthy cells are surrounded by a red circle, while the infected cells are surrounded by yellow and green circles, denoting the intra-erythrocytic stages trophozoite and schizont.

With our simple cell detection algorithm, we present a lightweight method to classify malaria-infected RBCs in microscopy images into four categories: healthy, ring-, trophozoite-, and schizont-stage parasites. Our approach is not limited to a certain microscopy technique but demonstrated to be universally applicable for fundamentally different imaging techniques, which was demonstrated via the high classification accuracy on images recorded with Giemsa-stained light microscopy, AFM, and fluorescence microscopy. The reduction of dimension by selecting characteristic features of the RBCs significantly boosts the speed and classification accuracy of the network. This simple concept can easily be applied for the classification of general objects. Given the rising number of techniques, successfully applied for the imaging of RBCs, the algorithm can be augmented for any method with high contrast, formatted as text or image file.

## Computational resources

The Malaria Stage Classifier is accessible as a GUI, which is arranged in tabs. It has been developed using Python 3.7 with the following dependencies: *numpy* [10] *and pandas* [11] for the data analysis. For the cell detection, *OpenCV* [12] together with *skimage* [13] and *matplotlib 3.5.2* [5] for the data visualisation are needed. To integrate the pre-trained NN, *tensorflow* [14] with *keras* has to be included in the algorithm. The interface further requires *tkinter* [15] and the libraries *os, sys, csv,* traceback for handling errors and the output files, as well as the library *webbrowser* to open system folders and external links.

Common errors are prevented by messages, detailing the problem and providing a solution. Furthermore, the errors saved in a log file to allow for bug-fixing by any developer. While the user is able to modify a few parameters through the GUI, suitable values are suggested for each case and all parameters can be set back to the default values.

## Availability and future directions

The Malaria Stage Classifier is deposited at the git hub repository https://github.com/KatharinaPreissinger/Malaria_stage_classifier or the archive https://zenodo.org/record/7261800. All details about the execution and usage of the package are documented in the documentation of our repository on https://github.com/KatharinaPreissinger/Malaria_stage_classifier or on https://malaria-stage-classifier.readthedocs.io/en/latest/index.html. There, we provide a tutorial for downloading the package and files with test images. The documentation includes a package for the stage-specific classification of RBCs (Malaria_stage_classifier.zip) with four folders: Code, Logo, Neural_networks, and Sample_images, which can be downloaded from the github repository https://github.com/KatharinaPreissinger/Malaria_stage_classifier. Our code contains four modules. NN.py initializes the neural network and trains the data. Classes.py contains classes for evaluating the properties of each RBC. Contours.py provides functions for the detection of RBCs in an image. ExtractCuts.py provides function for extracting the most characteristic profiles in the RBC. The folder also contains Malaria_stage_classifier, which runs the GUI. In Logo, the logo for the pop up window is located. If the researchers want to change the Networks, they can copy them to the Neural_newtorks folder, which also contains the pretrained networks. In sample images, some test images are provided. The program also allows for choosing any other image located on the computer used for analysis. The data set required for re-training the NN can be downloaded from https://zenodo.org/record/6866337.

Researchers are encouraged to implement new methods themselves, as the documentation is easily understandable and can be found at https://malaria-stage-classifier.readthedocs.io/en/latest/index.html. In case of low contrast microscopy images, the option of contrast improvement can be implemented, including thresholding for text files and the enhancement of brightness and contrast for image files. Moreover, we provide the opportunity to increase the data set, which is used to train the NN, to improve the classification accuracy of the algorithm. After loading the NN and visually verifying the accuracy of the classifications, the researchers have the possibility to add the characteristic single-RBC cross-sections to the original images and to retrain the NN with the new data set. The dataset for the new training is deposited at https://zenodo.org/record/6866337. We further encourage researchers in the malaria community to use our package to also train other malaria species, making our approach specific to both species and stages.

## Ethics statements

None

## CRediT authorship contribution statement

**Preißinger Katharina:** Conceptualization, Methodology, Software, Validation, Data curation, Writing – original draft, Visualization, Investigation. **Kézsmárki István:** Supervision. **Török János:** Conceptualization, Methodology, Software.

## Declaration of Competing Interest

The authors declare that they have no known competing financial interests or personal relationships that could have appeared to influence the work reported in this paper.

*Please declare any financial interests/personal relationships which may be considered as potential competing interests here.*

## Data Availability

I have shared the link to my data/code in the Attach File step.

## Appendix Supplementary materials

Image, application 1

## References

[1] Preißinger K. (2022). Reducing data dimension boosts neural network-based stage-specific malaria detection. Sci. Rep..

[2] Abbas S. (2020). Detection and stage classification of Plasmodium falciparum from images of Giemsa stained thin blood films using random forest classifiers. Diagn. Pathol..

[3] Otsu N. (1979). A threshold selection method from gray-level histograms. IEEE Trans. Syst. Man Cybern..

[4] F. Lundh, A. C. (2022). Pillow (PIL Fork) Reference. https://pillow.readthedocs.io/en/stable/reference/index.html.

[5] M.d team. (2022). Matplotlib 3.5.2 documentation. Von https://matplotlib.org/stable/index.html abgerufen

[6] J. Canny (1986). A computational approach to edge detection. IEEE Transactions on Pattern Analysis and Machine Intelligence, 679–698. 21869365

[7] Hough P. (1959). Proceedings of the International Conference on High Energy Accelerators and Instrumentation.

[8] Hubel D.H. (1968). Receptive fields and functional architecture of monkey striate cortex. J. Physiol..

[9] Prasanna H. (2007).

[10] Oliphant T. (2006).

[11] McKinney W. (2010). Proceedings of the 9th Python in Science Conference 1 (Scipy).

[12] T. OpenCV (2021). Hough circle transform. https://docs.opencv.org/3.4/d4/d70/tutorial_hough_circle.html.

[13] S.I.D. team (2022). Scikit-image: iamge processing in python. https://scikit-image.org/docs/stable/user_guide.html. 10.7717/peerj.453 PMC4081273 25024921

[14] NVIDIA. (2022). TensorFlow. https://docs.nvidia.com/deeplearning/frameworks/tensorflow-user-guide/index.html.

[15] P.S. Foundation (2022). *Graphical user interfaces with Tk*. https://docs.python.org/3/library/tk.html.

