# Supplementary material for: An automated neural network-based stage-specific malaria detection software using dimension reduction: The malaria microscopy classifier
Source: MethodsX. 2023 Apr 20;10:102189. doi: 10.1016/j.mex.2023.102189 (PMC10165163; doi:10.1016/j.mex.2023.102189)
Supplement: Supplementary file 1 [file mmc1.docx]

**Supplementary material *and/or* additional information**

**Malaria diagnosis - state of the art**

More ancient than human, malaria has caused millions of deaths until today (Ghebreyesus, 2021). Nowadays, the most common technique used worldwide is light microscopy of Giemsa-stained blood smears, which is time-consuming and heavily relies on human performance. To overcome these issues, computer-aided recognition of malaria-infected red blood cells (RBCs) has recently gained high attention.

As the number of cases is increasing due to the ongoing COVID-19 pandemic and climate change, the need for rapid and easily accessible recognition of the disease is rising. The infection is caused by five types of the *Plasmodium* genus, upon which *Plasmodium falciparum* causes the most deaths. Injected into the human body by a mosquito bite, the malaria parasites migrate to the liver cells, where they begin a phase of asexual reproduction, which is followed by the release of many thousands of merozoites. During their 48h cycle in the blood stream, the parasites invade healthy RBCs and mature through three main stages---the ring, the trophozoite, and the schizont stage---, while altering the morphological and optical properties of the host RBCs (K. Preißinger P. M., 2021) (I. R. Dave, 2017)*.*

These well-explored transformations of the infected RBCs provide the basis for neural network (NN) based stage-specific recognition of malaria-infected RBCs in blood smears (M. Poostchi, 2019) (Mehrjou, 2013) (M. C. Mushabe, 2013) (K. Torres, 2018) (I. R. Dave, 2017). While clearly demonstrating the potential of NNs in malaria diagnosis, these pioneering approaches still face problems, e.g., in terms of sensitivity to different malaria species and stages. The following studies illustrate the state-of-the-art in this field.

Based on recent advances in high-resolution imaging techniques applied for the analysis of malaria-infected RBCs, especially topographic imaging (E. Nagao, 2000) (H. Shi, 2013) (K. Preißinger P. M., 2021) and infrared nano-imaging (D. Perez-guaita, 2018), recent studies on unstained RBCs (K. Preißinger P. M., 2021) have demonstrated the applicability of NNs to analyse not only light microscopy images but also images recorded with other microscopy techniques. These works imply that, when combined with NN-based analysis, malaria diagnosis may be extended to other imaging methods. This provides the motivation for this work, that aims at boosting the performance of microscopy techniques in malaria diagnosis using NNs with input data of reduced dimension.

**User manual**

The Malaria Stage Classifier is available open source on git hub repository https://github.com/KatharinaPreissinger/Malaria_stage_classifier or the archive https://zenodo.org/record/7261800. In the following, the program structure is explained on a sample atomic force microscopy image. The interface of the package is built with five tabs, where each performs one step of the stage-specific classification of RBCs. Starting with the general settings, the algorithm only allows text or image files as input. Depending on the file type, the user can set the number of header lines the input text file contains. In case of the sample image, the value is set to three and confirmed by the button “Set file format”. As the program is designed for multiple imaging methods, the respective technique determines the possible actions on the input file. The settings are completed by selecting an output path and the output file type, see figure 10*.*

**

*Figure 10:* **Setting of initial parameters:** input image, its format, imaging method, memory location of the results

In the second tab, the input image is displayed to show the location and stage of the cells. A screenshot of the interface is presented in figure 11*.*

**

*Figure 11:* **Show image.** The input file is shown as image.

If the image was recorded by AFM, the next step requires thresholding to enhance its contrast. While the value can be adapted by the user, the algorithm suggests a pre-calculated number, as shown figure 12. In case of the light microscopy techniques, this step is skipped.

**

*Figure 12:* **Threshold image.** This tab provides the option to enhance the image contrast by thresholding (disabled for light and fluorescence microscopy)

The next tab triggers the cell detection algorithm. Depending on the size, contrast, and brightness of the image, the algorithm offers the option to control the detection by manually changing the calculation parameters and further allows contrast enhancement. Optionally, the parameters can be set back to the default value. During this step, the dimension of the input data is reduced to the two characteristic cuts to capture the strongest features associated with the presence or absence and the stage of the malaria parasites, see figure 13*.*

**

*Figure 13:* **Detect cells.** RBCs in the images are detected by the cell detection algorithm. To fine-tune, the algorithm offers the possibility to enhance the image and to set the parameters for the detection manually.

In the final part of the program, the user can load the pre-trained NNs and start the stage-specific prediction of the detected RBCs. This triggers the option to change false predictions accordingly. The algorithm further provides the possibility to add new data and to retrain each NN. The last step then returns the statistics of the analysed RBCs in form of a text file or table. The corresponding tab is shown in figure 14.


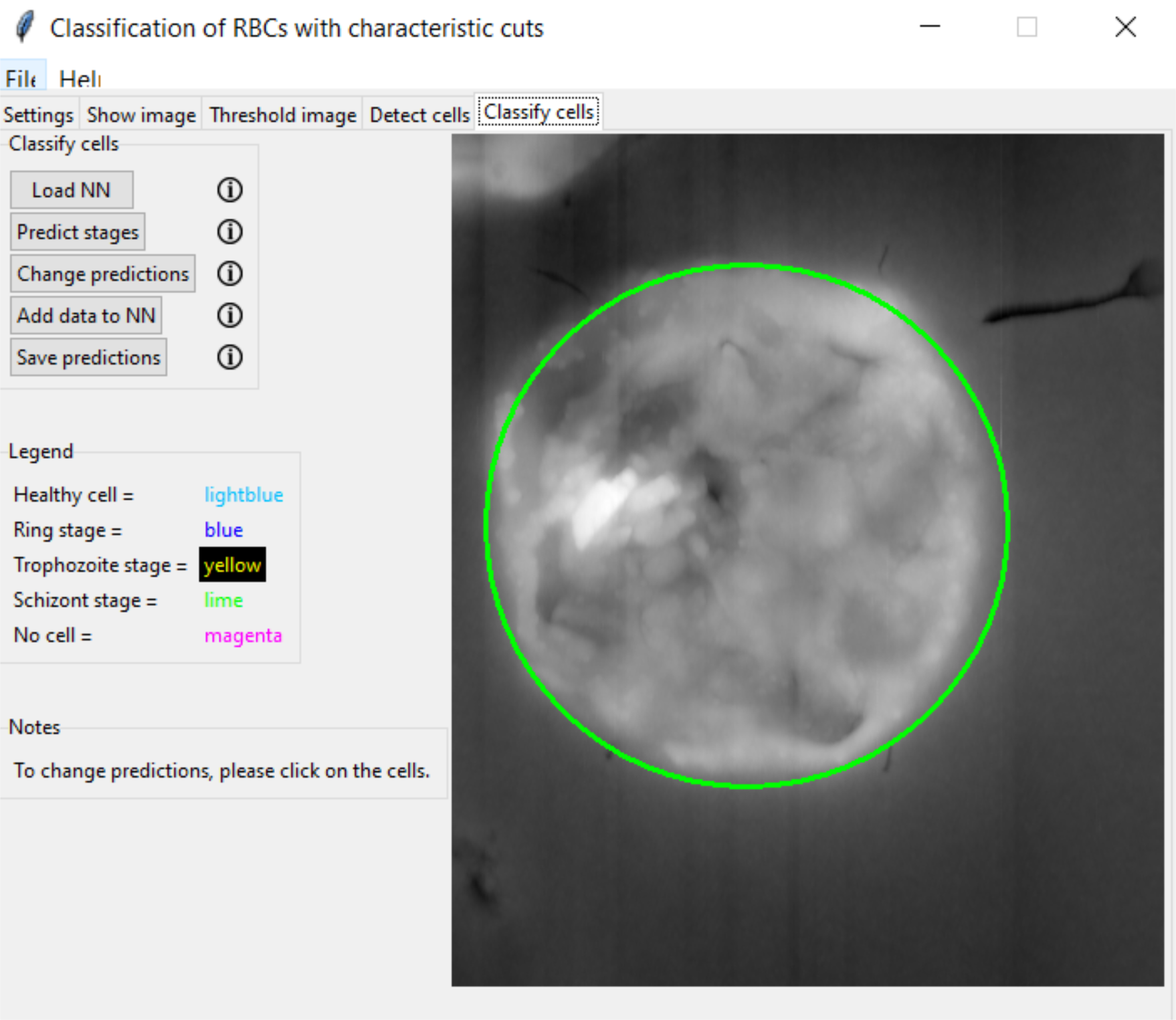


*Figure 14:* **Cell classification.** The intra-erythrocytic stages are predicted and can be altered manually. Optionally, the neural network can be retrained with new data

**Sample preparation**

Cultures of P. falciparum parasites from the laboratory adapted strain 3D7 were maintained in culture medium (Albumax, 25 mg/L Gentamycin, RPMI 1640) in an atmosphere of 5% CO2 and 5% O2 as in (Trager & J, 1976), (Schuster, 2002). The cultures were raised to > 5% parasitemia for each measurement and used to make a thin film smear on VWR microscope slides (90° ground edges, nominal thickness 0.8 - 1.0 mm).

**Morphological measurements**

To measure the morphology of RBCs on dried smears, we used an MFP-3D AFM (Asylum Research, Oxford Instruments) as in (K. Preißinger P. M., 2021), which was operated in AC mode at a scanning speed of 0.25 Hz. Each scan was performed on an area of 90 x 90 μm with a resolution of 512 x 512 pixels. The cantilever was an OTESPA-R3 from Bruker with a rectangular shape, a tip radius of 7-10 nm, a spring constant of 26 N/m, operated at a frequency of 280 - 300 kHz with a drive amplitude of 250 - 300 mV. The software used for the set up and acquisition of the AFM was Igor Pro 6.37.

**Fluorescence measurements**

The fluorescence microscopy images were obtained as in Ref. (K. Preißinger P. M., 2021) by using an Olympus IX81 inverted microscope, operated at a wavelength of 405 nm. Throughout the experiments, we used an Olympus TIRF objective (UApo N, 100x, 1.49NA). Our samples were excited by an OMICRON (Rodgau-Dudenhofe, Germany) LaserHub, which contained four independent laser sources (405 nm 120mW CW diode, 488 nm 200mW CW diode, 561 nm 156mW CW diode, 642 nm 140mW CW diode). The lasers were operated at 5 - 20% power controlled by the OMICRON Control Center software (v.3.3.19). An EM-CCD camera (Andor iXon DU-885KCSO-VP, Oxford instruments) collected images through a quad-band dichroic mirror and emission filter set (TRF89901-EM-ET-405/488/561/640, Chroma Technology, Bellows Falls, VT USA). The fluorescence measurements were carried out on RBC smears prepared on coverslips (nominal thickness 150 μm).
